# Revisiting the Concept of DIC: A Phenotype-guided Framework for Modern Hemostatic Medicine

**DOI:** 10.14789/ejmj.JMJ25-0066-R

**Published:** 2026-02-17

**Authors:** TOMAZ CROCHEMORE, ECATERINA SCARLATESCU

**Affiliations:** 1 Department of critical care, Hospital Moriah, São Paulo, Brazil; 1 Department of critical care, Hospital Moriah, São Paulo, Brazil; 2 Department of critical care, Hospital São Luiz Itaim, Rede Dor, São Paulo, Brazil; 2 Department of critical care, Hospital São Luiz Itaim, Rede Dor, São Paulo, Brazil; 3 Carol Davila University of Medicine and Pharmacy, Bucharest, Romania; 3 Carol Davila University of Medicine and Pharmacy, Bucharest, Romania; 4 Department of Anesthesia and Intensive Care III, Fundeni Clinical Institute, Bucharest, Romania; 4 Department of Anesthesia and Intensive Care III, Fundeni Clinical Institute, Bucharest, Romania

**Keywords:** disseminated intravascular coagulation, sepsis-induced coagulopathy, precision hemostatic medicine, coagulation biomarkers, viscoelastic testing

## Abstract

Disseminated intravascular coagulation (DIC) has long been described as a catastrophic systemic activation of coagulation with suppressed anticoagulant and fibrinolytic pathways, culminating in macro- and microvascular thrombosis, hypoperfusion, and multiple organ dysfunction. It may arise from acute inflammatory, traumatic, or infectious diseases, where consumption of fibrinogen, platelets, and coagulation factors, together with dysregulated fibrinolysis, lead to bleeding and poor outcomes.

Contemporary evidence demonstrates that DIC is not a single or uniform pathological entity, but rather the advanced and convergent phase of distinct coagulopathies triggered by heterogeneous systemic insults such as sepsis, trauma, burns, malignancy, and obstetric complications. Similar to acute respiratory distress syndrome and dialysis-dependent renal failure, organ-failure syndromes with different etiologies, DIC should be recognized as advanced failure of the coagulation system, characterized by excessive thrombin generation, impaired fibrin formation, depletion of endogenous anticoagulants, and fibrinolytic imbalance.

We propose reframing DIC as part of an etiologic and phenotype-guided framework within modern hemostatic precision medicine. The recognition of sepsis-induced coagulopathy (SIC), trauma-induced coagulopathy (TIC), and obstetric-associated coagulopathy (OAC), exemplifies this paradigm shift. Each condition exhibits unique inflammatory triggers, endothelial activation, and hemostatic trajectories evolving through identifiable phenotypes, from macrovascular hypercoagulability to microvascular fibrinolytic shutdown and, ultimately, to hypocoagulation, hyperfibrinolysis and hemorrhage.

Early identification of these phenotypes through viscoelastic testing and biomarkers such as D-dimer, fibrinogen, protein C, antithrombin, and plasminogen activator inhibitor-1 allows targeted, goal-directed interventions.

This strategy supports precision-guided anticoagulant therapy in thrombotic phenotypes and hemostatic resuscitation in hemorrhagic states, ultimately improving clinical outcomes in critically ill patients.

## Introduction: the obsolescence of a nonspecific syndrome

The term “disseminated intravascular coagulation” (DIC) emerged in the mid-20th century to describe systemic clot formation with concurrent bleeding. Diagnostic scores developed by the International Society on Thrombosis and Haemostasis (ISTH) formalized its laboratory criteria, focusing on platelet consumption, fibrin degradation products, and prolongation of prothrombin time^1)^. These criteria primarily capture end-stage, consumptive hypocoagulopathy, often after irreversible organ failure has begun and therefore fail to identify earlier, potentially reversible phases of the coagulopathy spectrum^2)^.

Recent evidence reinforces the concept of the coagulation system as a functional and adaptive organ, tightly interconnected with vascular, inflammatory, and immune pathways. Its alterations are graded, dynamic, and context-dependent, rather than a binary state of “normal versus DIC”^3)^. Instead of representing a single disease entity, DIC should be understood as a spectrum of pathophysiologic processes driven by the nature and intensity of the underlying insult^4)^. Consequently, labeling a patient as “overt DIC” offers limited therapeutic value, risks overlooking intermediate phenotypes, and may delay timely, mechanism-based intervention^5)^.

The evolution from DIC to induced coagulopathies mirrors paradigm shifts in other organ-failure syndromes^6, 7)^. Just as “acute respiratory distress syndrome” replaced the vague concept of “shock lung,” and “acute kidney injury” supplanted “uremic syndrome,” we now recognize that “DIC” is an umbrella term concealing disease-specific mechanisms that demand individualized treatment strategies^8)^.

This narrative review proposes an etiologic and phenotype-guided framework (SIC, TIC, CAC, OAC, and EIC) that replaces the generic label ‘DIC' with context-specific, induced coagulopathies and links these entities to distinct laboratory and clinical phenotypes amenable to targeted therapy^2, 9, 10)^. Each entity exhibits unique triggers, endothelial responses, and hemostatic trajectories that can be recognized in real time using viscoelastic testing (VET) and endothelial biomarkers (e.g., D-dimer, protein C, antithrombin, PAI-1). This precision approach underpins goal-directed hemostatic therapy, aiming to prevent progression to organ failure and reduce both thrombotic and hemorrhagic complications^2, 11-13)^.

## From DIC to a framework of pathology-induced coagulopathies （SIC, TIC, CAC, OAC, and EIC）

Among the many precipitants of coagulopathy, sepsis remains the most common and best characterized^2)^. In 2017, the concept of sepsis-induced coagulopathy (SIC) was introduced, and by 2019, it was formally adopted by the ISTH Scientific and Standardization Committee as the early phase of DIC^14, 15)^. The SIC score, derived from platelet count, PT-INR, and the Sequential Organ Failure Assessment (SOFA) score, enables early recognition of compensated coagulation disturbance before overt consumption occurs^15)^.

The same rationale applies to trauma-induced coagulopathy (TIC), ECMO-induced coagulopathy (EIC), cancer-associated coagulopathy (CAC), obstetric-associated coagulopathy (OAC), and other context-specific entities. Each begins with endothelial activation and tissue-factor release, followed by thrombin generation, suppression of natural anticoagulant pathways, and dysregulated fibrinolysis. Yet the magnitude, timing, and clinical expression of these events differ substantially:

• In sepsis, intense immune activation and glycocalyx degradation drive an early hypercoagulable and hypofibrinolytic phenotype^16)^;

• In trauma, shock-related hypoperfusion and tissue disruption precipitate an early and robust fibrinolytic surge, a hallmark of trauma-induced coagulopathy^17)^;

• ECMO support induces concurrent prothrombotic and consumptive mechanisms driven by blood-circuit interaction and systemic inflammation, resulting in a dynamic state of mixed hypo- and hypercoagulability^10)^;

• In obstetric complications, placental disruption, massive tissue-factor release, and endothelial injury precipitate abrupt hypercoagulability that may rapidly evolve into consumptive coagulopathy (e.g., PPH, abruption, amniotic fluid embolism)^18)^;

• In cancer-associated coagulopathy, tumor- related tissue factor expression, NETosis, and chronic endothelial activation sustain a persistent hypercoagulable and hypofibrinolytic state, increasing the risk of both macro- and microvascular thrombosis^19)^;

Recognizing these entities as induced, context- specific coagulopathies underscores why a single, late-stage term such as ‘DIC' is conceptually and therapeutically insufficient. By focusing on the causative trigger and hemostatic phenotype, clinicians can tailor therapy, anticoagulant, antifibrinolytic, or hemostatic, according to the stage of disease, rather than applying uniform treatment to all patients labeled as DIC^20)^.

## Dynamic pathophysiology: from thromboinflammation to hemostatic failure

Hemostasis operates as an adaptive defense mechanism designed to contain infection and restore vascular integrity. When dysregulated, this defense transforms into generalized thromboinflammation^21)^. The interaction between leukocytes, platelets, and the vascular endothelium drives the transition from localized protection to systemic injury^22)^.

**Initial phase:** Pro-inflammatory cytokines, such as TNF-α, IL-1β, and IL-6, trigger the expression of tissue factor in monocytes and endothelium, amplifying thrombin generation^23)^. Natural anticoagulant pathways including antithrombin, protein C, and tissue factor pathway inhibitor, are suppressed, while the endothelial glycocalyx begins to degrade^24)^. The result is macrovascular hypercoagulability characterized by elevated levels of D-dimer and fibrinogen, phenotype of thrombotic coagulopathy due to hypercoagulability^25)^.

**Intermediate phase:** Excess plasminogen activator inhibitor-1 (PAI-1) suppresses fibrinolysis, leading to fibrinolysis shutdown and microvascular thrombosis. Subsequent organ hypoperfusion manifests as early organ dysfunction induced by sepsis^26)^-phenotype of thrombotic coagulopathy due to fibrinolysis shutdown.

**Late phase:** Persistent inflammation leads to consumption of coagulation factors, fibrinogen and thrombocytopenia, characterizing a hypocoagulable state, associated with hemorrhagic manifestations-phenotype of hemorrhagic coagulopathy due to hypocoagulability. When PAI-1 stores are depleted, hyperfibrinolysis can be the final mechanism of coagulopathy-the phenotype of hemorrhagic coagulopathy due to hyperfibrinolysis^27)^. Bleeding becomes clinically significant at this terminal stage, the traditional “overt DIC”^28)^.

These phases correspond to discrete thrombotic, fibrinolytic-shutdown, and hemorrhagic phenotypes, which offer more actionable therapeutic targets than the unitary diagnosis of DIC. Therefore, what we have historically diagnosed as DIC represents only the final moment of a continuum that begins with endothelial activation and ends in hemostatic exhaustion. Recognizing the specific phenotype of each stage is essential for targeted therapy^29)^.

## Endothelial injury and the glycocalyx: the hidden origin of coagulopathy

The vascular endothelium, lined by a fragile glycocalyx composed of proteoglycans and glycosaminoglycans, maintains vascular homeostasis, providing an antithrombotic and anti-inflammatory surface^16)^. During sepsis or trauma, enzymatic degradation and oxidative stress disrupt this barrier, exposing adhesion molecules such as E-selectin and ICAM-1. This promotes leukocyte and platelet adhesion, microthrombus formation, and capillary extravasation^30)^.

Loss of glycocalyx integrity increases vascular permeability, promotes interstitial edema, and amplifies the systemic inflammatory response. Circulating glycocalyx degradation products, such as syndecan-1 and heparan sulfate, have emerged as biomarkers of endothelial injury and correlate strongly with disease severity and outcomes^31)^.

The destruction of this microvascular interface explains the heterogeneous coagulation profiles observed in sepsis. Although conventional coagulation tests (CCTs) may suggest the presence of hypocoagulation, recent studies show that VET allow differentiation between hyper- and hypocoagulability, and concomitant fibrinolytic imbalance^32)^. These observations support a shift from viewing coagulopathy purely as factor deficiency toward a paradigm in which endothelial integrity largely determines the hemostatic phenotype.

By integrating endothelial biomarkers with VET, physicians can monitor the transition from thrombotic to hemorrhagic states in real time, allowing phenotype-guided hemostatic management long before conventional DIC scores become abnormal^33)^.

## Clinical and laboratory evidence supporting the phenotype-based model

### Viscoelastic profiling: seeing beyond conventional coagulation tests

Conventional coagulation tests (CCTs), such as prothrombin time (PT), activated partial thromboplastin time (aPTT), fibrinogen concentration, provide static, plasma-based information that does not capture the cellular and dynamic features of in vivo coagulation^34)^. Viscoelastic assays (ROTEM/TEG) allow real-time assessment of clot initiation, propagation, firmness, and lysis, thus offering a more physiologically relevant picture of hemostasis^35)^.

Several studies have demonstrated marked discordance between CCTs and viscoelastic findings in critically ill and septic patients. In a large ICU cohort, more than half of critically ill patients exhibited normal viscoelastic profiles despite abnormal PT/aPTT or thrombocytopenia, which frequently prompted fresh frozen plasma (FFP) or platelet transfusion in the absence of viscoelastic evidence of hypocoagulability^36)^. This pivotal study underscores that coagulopathy defined by CCTs may not represent true hypocoagulability, and that viscoelastic monitoring can prevent inappropriate transfusion and improve individualized hemostatic care^37)^.

In a prospective cohort of 161 patients with sepsis or septic shock, 72.7 % demonstrated coagulopathy by ROTEM, subdivided into 25.5% hypercoagulable, 54.7% hypocoagulable, and 13.6% mixed patterns^38)^. Even among patients with prolonged PT/INR, viscoelastic analysis frequently revealed preserved or increased clot firmness, indicating ongoing thrombin generation and fibrin polymerization. FIBTEM- MCF strongly correlated with fibrinogen levels (ρ = 0.73), while EXTEM and INTEM parameters reflected platelet and thrombin-mediated effects^38)^.

Another observational study confirmed that patients labeled as “hypocoagulable” by CCTs often displayed latent hypercoagulability on ROTEM, particularly when fibrinogen was elevated^39)^. These findings illustrate that SIC is not unidirectional but fluctuates dynamically across thrombotic and hemorrhagic states.

Therefore, VET provides a real-time *window* into the evolving phenotype and should be used as a monitoring tool to tailor therapy, anticoagulation in early thrombotic phases and goal-directed hemostatic resuscitation in later hemorrhagic stages^40-43)^.

## Biomarker-guided phenotyping in sepsis-associated coagulopathy

### Thrombocytopenia

Thrombocytopenia (TCP) represents both a diagnostic and prognostic marker in sepsis^44)^. A progressive decline in platelet count reflects endothelial injury, increased platelet consumption through microvascular thrombosis, and immune-mediated destruction^45)^. Severe TCP (<100 × 10^9^/L) has been consistently associated with higher risk of septic shock and mortality^46)^. Importantly, platelet count changes, rather than single values, carry superior predictive power^47)^, as highlighted in the 2024 narrative review on sepsis-related TCP, which describes heterogeneous mechanisms ranging from cytokine- driven marrow suppression to macrophage-mediated clearance^48)^.

A British prospective multicenter observational study reported a 35.4% mortality rate among ICU patients with severe TCP, underscoring its role as a strong marker of critical illness severity^49)^.

Thrombocytopenia is highly prevalent in sepsis, affecting between 22% and 58% of septic patients, depending on disease severity and timing of assessment^50)^. Its mechanisms are multifactorial, including cytokine-mediated bone marrow suppression, peripheral consumption due to microvascular thrombosis, immune-mediated platelet destruction, hemodilution, and splenic sequestration^51)^. Clinically, TCP confers a dual risk profile: while severe reductions in platelet count predispose to bleeding, early microthrombus formation and impaired fibrinolysis can paradoxically sustain or even amplify hypercoagulability^25)^. This complex interplay explains why TCP consistently correlates with worse outcomes, including greater organ dysfunction, prolonged ICU stay, and increased mortality^52)^.

Against this complex background, the finding that approximately 18-20% of septic shock patients with TCP demonstrated hypercoagulability on TEG is particularly noteworthy^53)^. Despite reduced platelet counts, traditionally interpreted as a marker of hypocoagulability, a substantial subset of patients had preserved or even augmented clot strength. More importantly, hypercoagulability was independently associated with lower mortality (OR 0.395), suggesting that a maintained or compensatory coagulation response may reflect a more intact endothelial function and host-defense mechanism in early sepsis^53)^. This observation challenges the conventional assumption that TCP uniformly indicates bleeding risk and highlights the importance of phenotyping coagulation status rather than relying solely on platelet counts.

### Fibrinogen

Fibrinogen functions both as an acute-phase reactant and as the substrate for fibrin formation, resulting in a characteristic biphasic pattern with early elevation and late consumption in sepsis. Early in sepsis, fibrinogen levels may rise due to IL-6- mediated hepatic synthesis, but progressive consumption and hyperfibrinolysis can later result in hypofibrinogenemia^54)^. Low fibrinogen (< 1.5 g/L) marks the transition from thrombotic to consumptive phenotype and correlates with bleeding risk and mortality^55)^. Conversely, high fibrinogen levels in early sepsis are linked to hypercoagulability on VET , high clot firmness parameters, reflecting a compensatory phase prior to decompensation^38)^.

### D-Dimer

The D-dimer, a fibrin degradation product, remains one of the most widely used biomarkers of DIC^56)^. Elevated levels reflect ongoing fibrin formation and lysis, but the magnitude of elevation carries distinct prognostic implications. High D-dimer concentrations (> 4 mg/L FEU) are strongly correlated with septic shock and mortality^57)^. In a large pediatric ICU cohort (n = 7,648), D-dimer demonstrated the highest predictive performance for in-hospital mortality (AUROC = 0.77), surpassing PT, aPTT, and fibrinogen^58)^. Meta-analysis evidence confirms its independent prognostic value across age groups and clinical settings, making D-dimer a cornerstone biomarker for risk stratification in SIC^59)^.

### Protein C

Protein C (PC) has emerged as one of the most robust prognostic biomarkers in sepsis, with consistently lower levels observed in non-survivors compared to survivors^60)^. The systematic review and meta-analysis by Catenacci et al. demonstrated a significant standardized mean difference favoring higher PC levels in survivors (SMD 0.52, 95% CI 0.24-0.81), with similar patterns confirmed in 28- day mortality analyses (SMD 0.44, 95% CI 0.26-0.61)^61)^.

These data indicate that a decline in PC is associated with progressive endothelial dysfunction and a reduction in natural anticoagulant capacity. Serial measurement of PC could therefore serve as a dynamic marker of transition from compensated to decompensated coagulopathy^25)^.

Integrating PC levels, platelet count, PT-INR, and viscoelastic findings may improve risk stratification and help identify candidates for therapies aimed at restoring endothelial anticoagulant function, such as antithrombin or recombinant thrombomodulin^62)^.

### Antithrombin

Decreased antithrombin (AT) levels are one of the most consistent findings in sepsis and are strongly associated with the severity of organ dysfunction and mortality^63)^. The consumption of AT results from its continuous neutralization of thrombin and factor Xa, combined with impaired hepatic synthesis and degradation by neutrophil elastase^64)^. In severe sepsis, AT activity often drops below 70%, which correlates with disseminated microthrombosis and poor outcomes^65)^. Restoration of AT activity through substitution therapy has been linked to improved 28-day survival in patients with sepsis-associated DIC when anticoagulant therapy is guided by biomarker-based stratification^66)^.

### Plasminogen Activator Inhibitor-1 (PAI-1)

Among the fibrinolysis regulators, plasminogen activator inhibitor-1 (PAI-1) plays a pivotal role in defining the early thrombotic phenotype of SIC. Elevated PAI-1 levels inhibit both tissue- and urokinase-type plasminogen activators, suppressing fibrinolysis and promoting microvascular fibrin deposition^67)^. Multiple observational studies have demonstrated that high PAI-1 concentrations correlate with organ dysfunction, DIC progression, and mortality^68-70)^. In a multicenter cohort, PAI-1 levels ≥ 48.7 ng/mL predicted sepsis-associated liver dysfunction, while values ≥ 83 ng/mL were independently associated with 28-day mortality and DIC development^70)^.

A 2023 systematic review and meta-analysis encompassing over 4,000 patients confirmed that non-survivors exhibit significantly higher PAI-1 concentrations, establishing it as one of the most powerful independent biomarkers of severity and outcome in sepsis^71)^. From a mechanistic standpoint, PAI-1 links endothelial activation to fibrinolytic shutdown, bridging the transition from hypercoagulability to microvascular thrombosis^72)^. Therefore, serial PAI-1 assessment, combined with viscoelastic lysis indices, can delineate fibrinolytic phenotypes and guide timing of anticoagulant or fibrinolytic modulation in precision hemostatic management^26)^.

### Synthesis

Taken together, progressive TCP, declining fibrinogen, reduced PC and AT activities, accompanied by markedly elevated Ddimer and PAI1 concentrations, define a distinct biochemical and functional signature of advanced SIC^25, 73)^.

The integration of these biomarkers, rather than their isolated interpretation, captures the dynamic interplay between excessive thrombin generation, impaired fibrin formation, depletion of natural anticoagulants, and dysregulated fibrinolysis. When combined with viscoelastic assays, which provide real-time assessment of clot initiation, propagation, stabilization, and lysis within the cellular coagulation model, this composite hemostatic profile enables precise phenotyping of coagulopathy trajectories and the identification of optimal therapeutic windows for antithrombotic, fibrinolytic, or pro-hemostatic interventions^74)^.

This biomarker-driven and phenotype-guided strategy reflects a shift from the traditional descriptive view of DIC to a modern conceptual framework of pathology-induced coagulopathies, fully aligned with the principles of precision hemostatic medicine^32, 41, 75)^.

## Thrombotic and hemorrhagic phenotypes in critical illness

Recent expert consensus distinguishes two dominant clinical phenotypes of coagulopathy in critically ill patients^75)^:

1. **Thrombotic coagulopathies**, marked by systemic hypercoagulability and hypofibrinolytic states, typical of early sepsis, late trauma, or cancer.

2. **Hemorrhagic coagulopathies**, characterized by hypocoagulation and/or hyperfibrinolysis, observed in late-stage sepsis, obstetric catastrophe, early trauma or massive transfusion.

Patients often oscillate between these phenotypes according to disease evolution and therapeutic interventions^75)^.

The point at which patients transition from thrombotic to hemorrhagic phenotypes corresponds to hemostatic exhaustion and has traditionally been captured by the label ‘DIC', despite representing opposite physiologic states along a continuum.

Recognizing this continuum emphasizes the need to re-evaluate the term DIC: it merges contrasting physiological conditions into a single vague label and does not provide clarity for treatment options.

## Toward phenotype-guided hemostatic therapy

The management of coagulopathy should follow the principle of treating the mechanism, not the score. A phenotype-guided framework can be summarized as follows:

1. **Etiologic classification** — Identify the trigger: sepsis (SIC), trauma (TIC), ECMO (EIC), cancer (CAC), or obstetrics (OAC).

2. **Phenotypic definition** — Determine the hemostatic profile via VET, platelet function, fibrinogen, D-dimer, PC, AT and PAI-1:

•Thrombotic (hypercoagulable, fibrinolytic shutdown)

•Hemorrhagic (hypocoagulable, hyperfibrinolytic)

• Mixed/transition state.

3. **Targeted intervention**

• *Thrombotic phenotype*: consider heparin, antithrombin, or recombinant thrombomodulin; maintain normothermia and avoid procoagulant transfusions^76)^;

• *Fibrinolytic-shutdown phenotype*: explore modulation strategies (thrombomodulin, cautious fibrinolytic therapy)^29)^;

• *Hemorrhagic phenotype*: implement goal- directed hemostatic resuscitation guided by viscoelastic endpoints^11)^.

This phenotype-driven strategy integrates coagulation monitoring, pathophysiologic mechanisms, and individualized therapy to mitigate both thrombotic and bleeding complications.

## Conclusion: the epitaph of DIC and the rise of hemostatic precision medicine

Disseminated intravascular coagulation, once regarded as a discrete and universal clinical entity, is now recognized as the common final pathway of multiple induced coagulopathies. Persisting with the DIC label alone risks obscuring early pathophysiological transitions, delaying timely intervention, and reinforcing a reactive rather than anticipatory approach to hemostatic management.

A shift toward a phenotype-driven, etiology- based lexicon (e.g., SIC, TIC, EIC, CAC and OAC), acknowledges that what has been termed ‘DIC' is the common end point of diverse induced coagulopathies rather than a single disease and offers a more accurate and clinically actionable framework. Through dynamic coagulation system monitoring, integrating the thrombotic profile (D-dimer, fibrinogen, platelet count), endothelial dysfunction markers (PC, AT, PAI-1), and viscoelastic assays, clinicians can monitor the evolving hemostatic profiles in real time, identify the dominant and transitional phenotypes, and implement targeted interventions at the optimal therapeutic window.

This transformation signals the rise of hemostatic precision medicine, where coagulation is understood as a dynamic organ system rather than a static set of laboratory abnormalities.

Future research should validate phenotype-specific therapeutic algorithms, leverage machine-learning approaches for real-time prediction of phenotype transitions, and establish international registries to refine criteria for SIC, TIC, and related entities across heterogeneous populations.

## Data availability

This article is a narrative review and does not involve the generation or analysis of any original datasets. All information presented is derived from publicly available published studies.

## Author contributions

TC conceived the study, originated the conceptual framework, developed the phenotype-guided interpretation of disseminated coagulopathies, conducted the literature review, interpreted all scientific evidence, and was the major contributor in writing the manuscript. ES critically reviewed the manuscript for intellectual content and contributed to scientific refinement and structural clarity. All authors read and approved the final manuscript.

## Conflicts of interest statement

The authors declare that there are no conflicts of interest.
